# Mechanistic Insight into Oxidative Stress-Triggered Signaling Pathways and Type 2 Diabetes

**DOI:** 10.3390/molecules27030950

**Published:** 2022-01-30

**Authors:** Anju Singh, Ritushree Kukreti, Luciano Saso, Shrikant Kukreti

**Affiliations:** 1 Nucleic Acids Research Lab, Department of Chemistry, University of Delhi (North Campus), Delhi 110007, India; anju11278@gmail.com; 2 Department of Chemistry, Ramjas College, University of Delhi, Delhi 110007, India; 3 Genomics and Molecular Medicine Unit, CSIR-Institute of Genomics and Integrative Biology (IGIB), Mall Road, Delhi 110007, India; ritus@igib.in; 4 Department of Physiology and Pharmacology “Vittorio Erspamer”, Sapienza University of Rome, P. le Aldo Moro 5, 00185 Rome, Italy; luciano.saso@uniroma1.it

**Keywords:** oxidative stress (OS), ROS, diabetes mellitus (DM), antioxidants, insulin, mitochondria

## Abstract

Oxidative stress (OS) is a metabolic dysfunction mediated by the imbalance between the biochemical processes leading to elevated production of reactive oxygen species (ROS) and the antioxidant defense system of the body. It has a ubiquitous role in the development of numerous noncommunicable maladies including cardiovascular diseases, cancers, neurodegenerative diseases, aging and respiratory diseases. Diseases associated with metabolic dysfunction may be influenced by changes in the redox balance. Lately, there has been increasing awareness and evidence that diabetes mellitus (DM), particularly type 2 diabetes, is significantly modulated by oxidative stress. DM is a state of impaired metabolism characterized by hyperglycemia, resulting from defects in insulin secretion or action, or both. ROS such as hydrogen peroxide and the superoxide anion introduce chemical changes virtually in all cellular components, causing deleterious effects on the islets of β-cells, in turn affecting insulin production. Under hyperglycemic conditions, various signaling pathways such as nuclear factor-κβ (NF-κβ) and protein kinase C (PKC) are also activated by ROS. All of these can be linked to a hindrance in insulin signaling pathways, leading to insulin resistance. Hyperglycemia-induced oxidative stress plays a substantial role in complications including diabetic nephropathy. DM patients are more prone to microvascular as well as atherosclerotic macrovascular diseases. This systemic disease affects most countries around the world, owing to population explosion, aging, urbanization, obesity, lifestyle, etc. However, some modulators, with their free radical scavenging properties, can play a prospective role in overcoming the debilitating effects of OS. This review is a modest approach to summarizing the basics and interlinkages of oxidative stress, its modulators and diabetes mellitus. It may add to the understanding of and insight into the pathophysiology of diabetes and the crucial role of antioxidants to weaken the complications and morbidity resulting from this chronic disease.

## 1. Introduction

Myriad ailments such as neurodegenerative, cardiovascular and respiratory diseases and cancers are undoubtedly of major concern in present times. For decades, the lifestyle disease diabetes mellitus (DM) characterized by hyperglycemia and often associated with vascular complications has also become a major health issue. DM is a group of chronic metabolic disorders manifested in abnormal insulin secretion, insulin deficiency and insulin insensitivity. It is characterized by hyperglycemia which is associated with altered insulin secretion, leading to morbidity, dysfunction and failure of the normal functioning of vital organs, especially the eyes, liver, kidney, nerves, heart, etc. [1]. According to the WHO’s fact sheets on diabetes, in 2019, diabetes was the ninth leading cause of death, with an estimated 1.5 million deaths directly caused by diabetes. It was hypothesized in the metabolic theory of diabetes mellitus that complications such as endothelial as well as cellular deterioration arise owing to hyperglycemia, whereas genetic theory indicated that diabetes-related complications are predetermined genetically [2].

DM is classified largely into two broad categories, namely, type 1 DM and type 2 DM, where type 1 DM is an autoimmune disorder which harbors the alleles of the human leukocyte antigen (HLA) class II genes residing in the major histocompatibility complex (MHC). Type 1 DM is also known as insulin-dependent diabetes, where insulin secretion is deficient owing to the autoimmune destruction of β-pancreatic cells, leading to metabolic disorders. It has been found that approximately 5–10% of DM patients belong to type 1 DM, whereas 90% fit into type 2 DM. Almost all patients with type 1 DM have been observed to have an elevated level of autoantibodies (type 1A DM), while 10% of type 1 DM patients do not have serum autoantibodies. This condition is also known as maturity onset diabetes of the young (MODY), which is linked to the dysfunction of β-cells with autosomal dominant inheritance (type 1B DM) [3]. β-cell destruction ends in infiltrating monocytes, lymphocytes and a mixture of pseudoatrophic islets, along with the secretion of somatostatin, glycogen and pancreatic polypeptide, leading to the diabetes via an immunogenic process. The morbid effect of type 1 DM on health is manifested in damage of tiny blood vessels in the eyes, nerves, kidneys, heart, etc.

Type 2 DM significantly results from impaired insulin secretion by the pancreatic β-cells along with dysfunction in insulin action via insulin resistance. Type 2 DM is the more prevalent subtype and is confined to defective insulin secretion via the absence of inhibitory feedback through plasma glucagon levels, chronic exposure to free fatty acids, lipotoxicity, etc. [4,5,6]. Significant deterioration in glucose tolerance observed in type 2 DM is followed by a reduction in the pancreatic β-cell mass with insulin resistance. Obesity has also been found to be associated with type 2 DM which results in cellular oxidative stress and insulin resistance [7,8]. Characteristic symptoms of diabetes are polyphagia (increased hunger), polydipsia (increased thirst), polyuria (urge to urinate), etc. Hyperglycemia is found to play a crucial role in the malfunctioning and dysfunction of many vital organs such as the heart, kidneys, nerves and eyes [9]. Hyperglycemia specifically targets and damages vascular endothelial cells. The close link between diabetes and premature vascular disease is very well established [10,11].

Intriguingly, as with a number of health disorders such as cancer and cardiovascular (CVD) and neurodegenerative diseases, oxidative stress (OS) is found to be linked with diabetes mellitus (DM) [2,12,13,14]. OS-linked diabetes leads to the stimulation of the polyol pathway and advanced glycation end product (AGE) formation and induces the activation of protein kinase C (PKC), ultimately resulting in the production of reactive oxygen free radical species [15,16]. An elevated level of F2-isoprostane has been reported in the plasma of type 2 diabetes mellitus patients, and in the urine of type 2 as well as type 1 DM patients [17,18]. Glycemic impairment and enhanced lipid peroxidation are found to be closely linked [18]. A substantially increased level of antioxidants was observed in saliva and blood samples of DM patients, and this was considered as a counter effect of the elevation in free radical species leading to oxidative stress [19]. Overproduction of ROS has led to the suppression of non-enzymatic antioxidants and antioxidant enzymes in various tissues, causing enhanced OS in chronic hyperglycemic conditions [20,21,22,23]. A number of molecular pathways and factors play a crucial role in the induction of OS, which tends to upregulate diabetes. This review is a modest attempt to focus on the role of OS in the pathogenesis and complications of diabetes as well as the pivotal role of antioxidants in the prevention of the morbid effect of OS in diabetes.

## 2. The Malicious Effect and Complications of Diabetes Mellitus (DM)

Diabetes plays a crucial role in morbidity and mortality and has a significant economic cost to society. People with diabetes have a tendency to suffer from acute metabolic disorders such as hyperglycemic hyperosmolar nonketotic coma, diabetic ketoacidosis and hypoglycemia [24,25]. The malicious effect of diabetes can be separated into microvascular (involving small vessels) and macrovascular (including large vessels such as arteries and veins) effects. While microvascular ailments concern diabetic nephropathy, retinopathy and neuropathy, macrovascular disorder is found to play a vital role in coronary artery disease, peripheral arterial disease and stroke (heart attack) [26,27]. Diabetic nephropathy is involved in end-stage renal disease which involves a decline in the glomerular filtration rate and in glomerular and tubular epithelial hypertrophy and elevation in urinary albumin excretion, along with mesangial expansion with the accumulation of extracellular matrix proteins (ECM) [28]. Glomerular filtration alteration ultimately results in abnormal albuminuria which can progress through a number of stages such as normal albuminuria, microalbuminuria and macroalbuminuria, resulting in end-stage renal disease [29].

Further, diabetic retinopathy is directly associated with visual impairment worldwide, which concerns damage of the small vasculature of the retina as well as light-sensitive tissue, at the back of the eye [30,31]. Diabetic neuropathy is characterized by a progressive loss of nerve fiber function along with deterioration in sensory nerves and damage to motor nerves. This happens owing to the cellular damage to endothelial cells, inhibition in nerve blood flow and damage to the neurons, leading to the conductivity of impulses [32,33]. It is manifested in symptoms such as the absence of sweating, numbness or tingling, having a burning sensation and weakening of reflexes [34].

**Figure 1:**
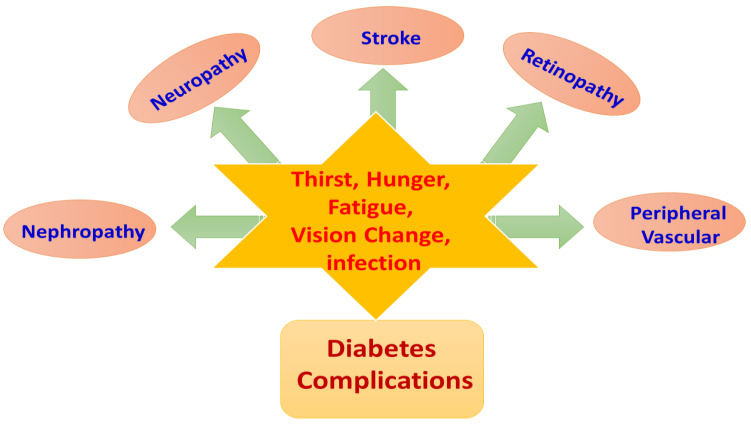
The symptoms of diabetic conditions and associated complications.

The literature is rich in reports that diabetes (type 1 and type 2) directly plays a central role in morbidity related to heart diseases such as coronary artery disease (CAD), stroke and peripheral arterial disease [35,36,37]. Patients suffering from diabetes have a significantly higher risk of cardiovascular disease (CVD), and approximately 80% of diabetes-associated mortality occurs due to cardiovascular disease. Excessive accumulation of lipids, cholesterol, inflammatory cells and connective tissue in the vessel wall leads to atherosclerosis, causing CVD-associated deterioration of health, and death [38,39]. Atherosclerotic plaque deposition in vessel lumina obstructs the flow of blood, resulting in cessation of heart tissues. The increased levels of free fatty acids under hyperglycemic conditions along with insulin resistance play a vital role in vascular dysfunction as well as the atherosclerotic process [40]. Alterations include an elevation in oxidative stress, a reduction in the bioavailability of NO and a hindrance in intracellular signal transduction, along with enhancement in the generation of many prothrombotic factors [41]. Figure 1 depicts the symptoms of diabetic conditions and associated complications.

## 3. Oxidative Stress, Reactive Free Radical Species and their Malicious Role played in Diabetes

Some three billion years ago, when the atmosphere was virtually devoid of oxygen, prokaryotic organisms anaerobically involved in photosynthesis activity usually relied on sulfur-based redox chemistry [42]. Later, cyanobacteria facilitated the production of oxygen via photosynthesis, raising the oxygen level in the environment, leading to symbiotic growth of organisms into mitochondria containing eukaryotic cells. These organisms were able to exploit oxygen for enormous energy production, required for survival and life sustainability [43]. In the case when any infection, disease, toxin or metabolic disorder occurs in the cellular environment, mitochondria generate reactive oxygen species (ROS) and reactive nitrogen species (RNS), leading to a reduction in oxygen consumption. This shielding acts as a defensive mechanism in the cellular environment either by lowering the cellular uptake of toxic substances or pathogens from the environment, or by inhibiting the spread of toxicity to the neighboring cells by apoptosis or cell death. Hence, the generation of ROS/RNS at an optimum level in biological systems is a normal physiological process to combat cellular stress.

**Figure 2:**
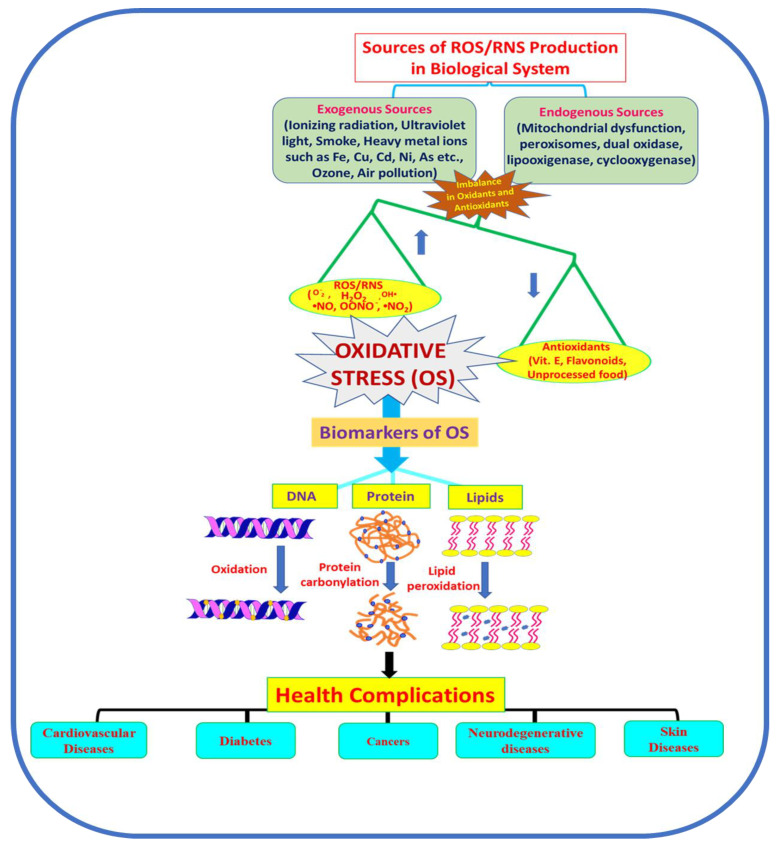
Schematic representation of some sources involved in the production of ROS/RNS, causes of oxidative stress, biomarkers of OS and diseases.

In general, the term “oxidative stress” is introduced to define a state between elevated levels of ROS/RNS and a decreased level of antioxidants causing a morbid effect in the cellular machinery. The source of production of these reactive species can be either exogenous (ionizing radiation, ultraviolet light, smoke, heavy metal ions such as Fe, Cu, Cd, Ni and As, ozone, air pollution, etc.) or endogenous (mitochondrial dysfunction, peroxisomes, dual oxidase, lipoxygenase, cyclooxygenase) [13]. Being free radicals, ROS and RNS, with their available electrons, react with other biomolecules such as lipids, proteins and nucleic acids (DNA and RNA). The duality of free radical species in biological systems signifies that, at an optimum level, they play a crucial role in cellular processes such as defense against infectious agents, induction of a mitogenic response and cellular structure maturation, whereas their elevated level causes a morbid effect [44,45,46]. Reactive oxygen species include the superoxide anion (O_2_^•^^−^), hydroxyl radical (•OH), hydrogen peroxide (H_2_O_2_) and hypochlorous acid, and reactive nitrogen species include nitric oxide (•NO), nitrogen dioxide (NO_2_^•^^−^) and peroxynitrite (OONO^−^). Elevation in these ROS/RNS leads to oxidative stress and ultimately results in deleterious effects on cells [47,48]. These free radical species can be produced either from exogenous (ionizing radiation, ultraviolet light, smoke, heavy metal ions such as Fe, Cu, Cd and Ni, etc.) or endogenous sources (mitochondrial dysfunction, peroxisomes, dual oxidase, lipoxygenase, cyclooxygenase, etc.). Figure 2 depicts the sources of ROS/RNS, OS and the malicious effect on biomolecules leading to various diseases.

The large amount of evidence of OS biomarkers in both diabetic patients and in rodents highlights the close link between oxidative stress and diabetes. In a hyperglycemic condition, an elevation in oxidative DNA damage markers such as 8-hydroxy-2′-deoxyguanosine (8-OHdG) and 8-oxo-7,8-dihydro-2′-deoxyguanosine (8-oxodG), lipid peroxidation products, thiobarbituric acid reactive substances (TBARS) and protein oxidation products (nitrotyrosine and carbonyl levels), along with reduced antioxidant enzyme activity, is observed. Numerous reports based on cell cultures using β-cells (liver), aortic smooth muscle cells and endothelial cells revealed that the ROS level is elevated in diabetic conditions [49]. Various pieces of experimental evidence of OS leading to morbid effects on many organs are tabulated in Table 1.

Free radical species are very efficient in oxidizing biomolecules (DNA, proteins, lipids, etc.); thus, they are thought to play a vital role in the onset and progression of late diabetic complications. The oxidative stress condition crops up when an elevation in ROS/RNS weakens the antioxidant defense mechanism, activating stress-sensitive intracellular signaling pathways. Stressed signaling trails result in the formation of gene products inducing cellular damage [50,51,52].

## 4. Metabolic Pathways Significantly Involved in Free Radical Generation in Diabetes and Resultant Complications

**Figure 3:**
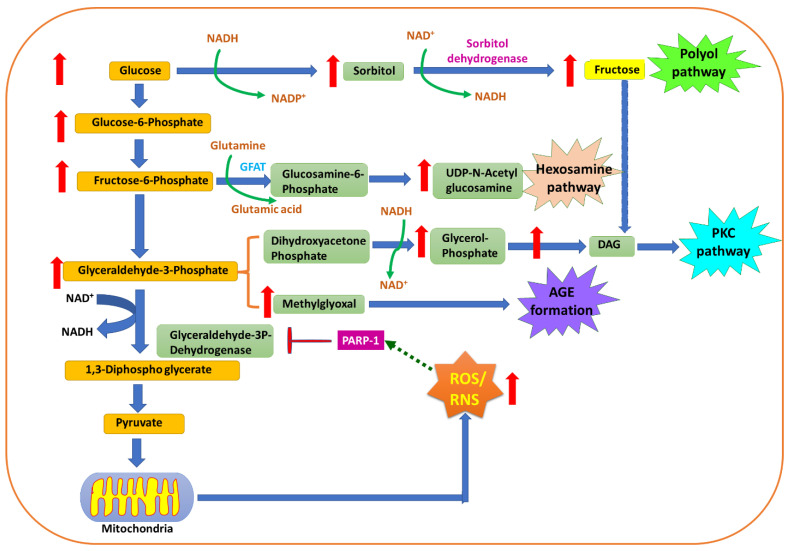
Pathways (glucose metabolism) and products which mediate oxidative stress.

The knowledge acquired thus far has suggested the mechanisms/pathways associated with hyperglycemia-induced diabetes and its complications. The associated damage majorly focuses on five pathways, such as an increased flux of glucose and other sugars through the polyol pathways [73], increased intracellular formation of advanced glycation end products (AGEs) [74], increased expression of the receptors for advanced glycation end products and their activating ligands, activation of protein kinase C (PKC) isoforms [75,76,77] and overactivity of the hexosamine pathways. It is believed that these pathways play crucial roles in the overproduction of ROS/RNS in the cellular environment, leading to OS and associated diabetic complications. Figure 3 demonstrated the pathways and products mediating the oxidative stress.

### 4.1. Glucose Oxidation and Glyceraldehyde-3-Phosphate Dehydrogenase (GAPDH)

It is an established fact that in order to generate energy for biological functions, glucose is exploited and oxidized inside the cells by the cellular respiration process. On this biochemical journey, the end product of glycolysis, a three-carbon compound, pyruvate, enters mitochondria and is converted to acetyl-CoA before it enters the Krebs cycle and becomes involved in mitochondrial metabolism. The byproducts of normal mitochondrial metabolism generate potentially damaging levels of ROS. ROS/RNS play a crucial role in inhibiting the enzyme glyceraldehyde-3-phosphate dehydrogenase via the activation of the enzyme poly-ADP-ribose polymerase-1 (PARP-1) [73]. PARP-1 is specifically involved in DNA repair and cell apoptotic pathways. PARP-1 is activated by the induction of strand breaks in nuclear DNA through the action of ROS/RNS. PARP-1 activation leads to the inhibition of GAPDH by poly-ADP-ribosylation. Glyceraldehyde-3-phosphate accumulation in cells is involved in the activation of two processes which mediate hyperglycemia complications, i.e., either it activates the AGE pathway by dragging glyceraldehyde-3-phosphate and dihydroxyacetone phosphate into the non-enzymatic synthesis of methylglyoxal, or the elevated level of glyceraldehyde-3-phosphate facilitates diacylglycerol production which, in turn, activates the PKC pathway. Further, levels of the glycolytic metabolite fructose-6-phosphate are elevated and increase the flux through the hexosamine pathway, where fructose-6-phosphate is converted to UDP-N-acetylglucosamine via the action of the enzyme glutamine-fructose-6-phophate amidotransferase (GFAT). Thus, this leads to the obstruction of GAPDH, in turn enabling the accumulation of glucose. This whole process enhances its flux via the polyol pathways, consuming NADPH in the process.

### 4.2. The Polyol Pathway

Under normal conditions, to enter the glycolytic pathway, cellular glucose predominantly undergoes phosphorylation to form glucose-6-phosphate by hexokinase. Only trace amounts of non-phosphorylated glucose (~3%) enter the polyol pathway. The aldo-keto reductase enzyme, the main rate-limiting enzyme of the polyol pathway, is involved mainly in catalyzing the reduction reaction of various carbonyl compounds into their respective alcohols. Aldose reductase is a key enzyme which plays a crucial role in catalyzing the nicotinamide adenine dinucleotide phosphate (NAD(P)H)-dependent reduction of glucose to sorbitol. Further, this reduction is followed by oxidation of sorbitol to fructose via an NAD^+^-dependent sorbitol dehydrogenase-mediated reaction. Aldo-keto reductase has a very low affinity for the normal glucose concentration (5.5 mM); thus, only a small amount of glucose can be metabolized by this pathway [78]. However, under hyperglycemic conditions, hexokinase saturation takes place, resulting in an elevation in glucose and entry to the polyol pathway. In diabetes, an elevation in polyol pathways takes place in tissues where insulin is not mandatory for cellular glucose uptake, such as the retina, kidney and peripheral nerves [73,79].

These abrupt reactions of the polyol pathway generate a reductive imbalance owing to the reduction in intracellular NAD(P)H, along with an elevated level of NADH, which acts as a substrate for the enzyme NADH oxidase to generate ROS/RNS. The reduced level of NAD(P)H crucially affects the antioxidant system by depleting the antioxidant glutathione (GSH) level in the cellular environment because the activity of GSH reductase significantly depends on NAD(P)H. At an optimum level of NAD(P)H, GSH reductase produces GSH from its oxidized form GSSH. The decreased level of NAD(P)H also affects the synthesis of nitric oxide (NO), known as a vaculoprotective agent. Nitric oxide synthase (NOS) in the presence of NAD(P)H synthesizes NO from L-arginine; here, NAD(P)H plays a key role as a cofactor for NOS. In the absence of a substrate or cofactor, endothelial nitric oxide synthase (eNOS) is involved in the production of the superoxide radical (O_2_^•^^−^) in place of NO, and this condition is known as the “uncoupled state of nitric oxide” [80].

Nitric oxide is thought to play an essential role in many important physiological processes such as vascular relaxation and inhibition of platelet activation and acts as an anti-inflammatory agent via obstructing platelet aggregation and adhesion. These actions, in turn, inhibit atherogenesis and protect blood vessels [81,82]. A remarkable dip in the bioavailability of NO will thus lead to an increase in inflammation as well as thrombosis and disturb the integrity of endothelial cells. Superoxide anions are quenched by NO straightaway, via the formation of the highly reactive peroxynitrite (OONO^−^). This mediates the initiation of lipid peroxidation and oxidizes the sulfhydryl group in proteins, nitrates and amino acids such as tyrosine, leading to a morbid effect on many signaling pathways. In the retina, the main pathway acting as a source of production of ROS is the polyol pathway [83]. Additionally, accumulation of sorbitol is found to play a malicious role in osmotic swelling of the eye lens, causing cataractogenesis [84].

### 4.3. Advanced Glycation End Products (AGEs)

Schiff base formation, in any cellular event, takes place via the reaction of glucose with free amino groups of proteins. The resultant Schiff bases undergo many complex reactions such as Amadori rearrangement, dehydration and condensation reactions producing heterogenous fluorescent derivatives known as advanced glycation end products (AGEs). The primary initiating event where both intracellular and extracellular AGEs are generated is recognized as intracellular hyperglycemia [85]. Intracellular reactions which involve the production of AGEs are as follows: auto-oxidation of glucose to glyoxal, decomposition of the Amadori product (glucose-derived 1-amino-1-deoxyfructose lysine adducts) to 3-deoxyglucosone and non-enzymatic phosphate elimination from glyceraldehyde phosphate and dihydroxyacetone phosphate to form methylglyoxal. Three general mechanisms of cell damage which can be mediated by AGE precursors are as follows: (i) AGE-modified intracellular proteins which can alter cellular function, (ii) AGE precursor-modified extracellular matrix components which interact abnormally with other matrix components as well as with matrix receptors (integrins), expressed on the cell surface, and (iii) AGE-modified plasma proteins that bind to AGE receptors such as RAGE and AGE-R1, 2 and 3 on the cells, i.e., macrophages, vascular endothelial cells and vascular smooth muscle cells. The production of ROS/RNS is induced via AGE receptor binding, leading to activation of PKC (along with activation of NF-κB (nuclear factor κB)) and NADPH oxidase, in turn causing morbidity in MAPK (mitogen-activated protein kinase) signaling [86].

### 4.4. Hexosamine Pathway

In normal cellular conditions, when glucose levels are in the normal range, a low amount of fructose-6-phosphate is moved away from glycolysis. However, under hyperglycemic conditions, the level of intracellular glucose is elevated; a greater amount of fructose-6-phosphate is moved out from glycolysis to facilitate the substrate for the enzyme glutamine: fructose-6-phosphate amidotransferase (GFAT), a rate-limiting enzyme of the hexosamine pathway. An elevation in the amount of fructose-6-phosphate results in the upregulation of GFAT activity. GFAT mediates the conversion of fructose-6-phosphate to glucosamine-6-phosphate [87]. Glucose-6-phosphate ultimately inhibits the glucose-6-phosphate dehydrogenase activity, which is the key enzyme involved in maintaining the level of NADPH. In diabetic conditions, mitochondrial ROS (superoxide) are elevated, which inhibits GAPDH activity, resulting in the accumulation of glycolytic intermediates. This series of events leads to an enhanced flux along the hexosamine pathway owing to the increased fructose-6-phosphate levels [88]. It has already been reported that the enhanced hexosamine pathway flux is a substantial non-mitochondrial source of ROS in the diabetic heart. It has also been observed that the level of hexosamine is enhanced in retinal tissues of humans as well as rats suffering from diabetes and might play a crucial role in toxicity linked with high glucose levels and ROS in cells [89].

### 4.5. Diacylglycerol Formation and PKC Activation

The protein kinase C (PKC) family is the largest kinase family, consisting of approximately 11 isoforms of serine/threonine kinases, which are crucially involved in signaling pathways activated by phosphatidyl serine, calcium and diacylglycerol (DAG). PKC activation plays a pivotal role in the progression of diabetes mellitus via vascular cell dysfunction as PKC activation is associated with vasoconstriction, proliferation and overgrowth of smooth muscle cells and enhanced synthesis of extracellular matrix proteins. Overexpression of PKC isoforms takes place via de novo synthesis of diacylglycerol (DAG) from glucose, with an increase in the availability of triose phosphate [90,91]. It is well documented that AGEs and their cell receptors are interlinked with an enhanced activity of PKC isoforms [92,93].

Complications in diabetes can be regulated by PKC at multiple levels such as via activation of eNOS, NAD(P)H oxidase, phospholipase A_2_ (PLA_2_), endothelin-1 (ET-1), vascular endothelial growth factor (VEGF), transforming growth factor-β (TGF-β) and NF-κB. PKC also mediates hindrances in the gene expression of key proteins and results in reduced blood flow, inflammation, occlusion of capillaries and free radical generation, leading to damage of cellular macromolecules [94]. PKC-dependent activation of NAD(P)H oxidase can lead to stimulation of ROS/RNS production by an increased level of glucose [90]. Interestingly, NAD(P)H is significantly found in phagocytic cells and is found to be the main source of ROS/RNS in non-phagocytic cells such as mesangial cells, endothelial cells, fibroblasts, podocytes and smooth muscle cells [95,96,97,98]. NAD(P)H oxidase-mediated generation of ROS/RNS may have a malicious effect on DNA in diabetic renal tissue, resulting in the development of nephropathy [99].

## 5. Insulin Resistance, Insulin Secretion in Diabetes and Effect of OS on These Processes

### 5.1. Role of OS in Insulin Action and Resistance

Insulin plays a crucial role in metabolism by exerting anabolic actions via its transportation to peripheral tissues. Insulin binds to a transmembrane protein belonging to the protein tyrosine kinase activity receptor superfamily, having the ability to autophosphorylate. This autophosphorylation, in turn, initiates a chain of reactions which are significantly involved in the activation of signaling pathways such as PI3K, MAPK and Cb1. These signaling pathways act collectively to translate the insulin receptor aroused signal into biological actions in target organs. These signals include glucose transport by transporting GLUT4 vesicles to the membrane, protein, lipid and glycogen synthesis and mitosis and ultimately deal with insulin gene expression [100,101].

**Figure 4:**
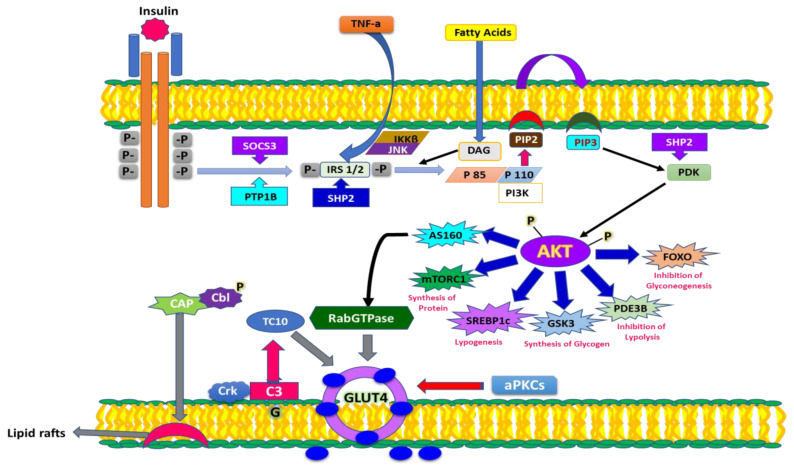
Mechanism of insulin action and factors involved in signaling.

It is evident that signaling pathways are activated via protein phosphorylation, and dephosphorylation inhibits them. A number of phosphatases (protein-tyrosine phosphatase 1B (PTP1B), phosphatase and tensin homolog (PTEN), SH2-containing tyrosine-protein phosphatase (SHO2) and suppressor of cytokine signaling 3 (SOCS-3)) play crucial roles in shutting down insulin signaling via dephosphorylation. Thus, inefficient phosphorylation as well as elevation in phosphatase activity causes a hindrance in insulin action. This, in turn, leads to insulin resistance. A schematic representation of insulin action and signaling is shown in Figure 4.

**Figure 5:**
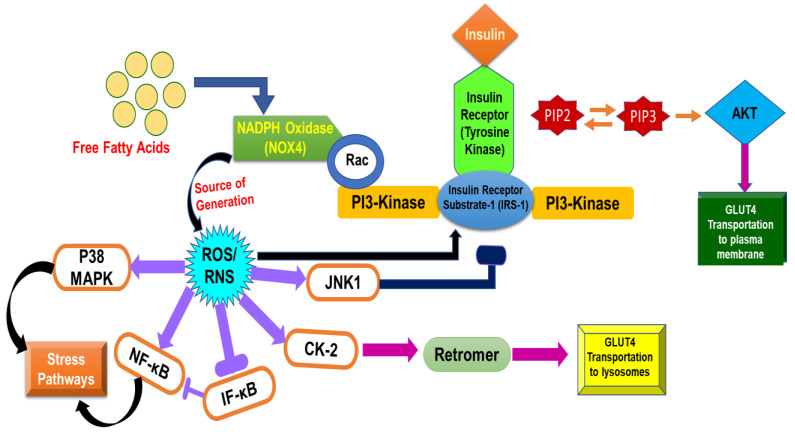
Normal pathway of insulin signaling and inhibitory effect of ROS in insulin signaling.

Insulin signaling is significantly affected by ROS/RNS. However, an optimum level of ROS/RNS is essential for proper insulin signaling, whereas an elevation in ROS/RNS can cause a malicious effect on insulin signaling. Insulin receptor stimulation takes place in adipocytes, which, in turn, regulate H_2_O_2_ production via NADPH oxidase. An elevation in insulin concentrations triggers the shift in the signaling pathway at P13-kinase. Aggravation in the activity of NOX4 is caused by the abrupt signaling of P13-kinase, which phosphorylates Rac (Rac GTPase) instead of PIP2 (phosphatidylinositol species), consequently leading to an elevation in ROS/RNS in the cellular environment [102]. Elevated ROS/RNS then activate casein kinase-2 (CK2) which, in turn, triggers the activation of retromer [103]. Retromer plays a role in the signaling of the trans-Golgi network, resulting in transportation of GLUT4 into lysosomes for degradation in place of being transported to the plasma membrane. This signaling contributes to an elevated level of glucose in the intravascular system, experiencing an oxidative stress condition. Figure 5 displays insulin signaling in normal conditions as well under the influence of ROS/RNS.

Mitochondria, the powerhouse of the cell, contribute crucially to oxidation in the cell owing to the high-nutrient environment. Intake of high-sugar supplements in the diet facilitates many substrates that are available for mitochondria to produce a substantial amount of ATP. Subsequently, mitochondria become hyperactive, and overproduction of free radicals takes place. The increased level of free radical species, in turn, deteriorates the cellular machinery and induces stress in the cellular environment [104]. Free radical species are directly involved in the stimulation of NF-κB (nuclear factor κB), JNK (c-Jun N-terminal Kinase) [105] and p38 MAPK [106], resulting in a mitochondria-induced stress response. The mitochondrial fission process is conducted by elevated free radical species affecting the functioning of the insulin receptor pathway as well as stress protein actions [107]. It is established that mitochondrial fission is directly linked to insulin resistance in skeletal muscles [108]. Several cell studies have demonstrated that insulin resistance can be inhibited by restricting overactivation of mitochondria [109].

### 5.2. Role of OS in Insulin Secretion

Insulin is secreted by β-cells of pancreatic islets of Langerhans in response to an elevation in glucose levels, known as glucose-stimulated insulin secretion. Basal and peak insulin levels are closely related to glucose concentrations. Glucose sensing and its metabolism take place via β-cells. This process involves two phases of insulin secretion: In the first phase of insulin secretion, glucose enters the cell with the help of the glucose transporter GLUT1 (in rodents GLUT2). This glucose is then phosphorylated to glucose-6-phosphate, mediated by glucokinase (hexokinase) [110]. It is a well-known fact that when glucose enters the cell and becomes metabolized, the ATP/ADP ratio is enhanced, which leads to the closure of K_ATP_ channels. These channels act as metabolic sensors, i.e., their opening and closing are entirely dependent on the ATP/ADP concentration in the cell. K_ATP_ closure facilitates slow depolarization of the membrane potential and enhances the opening probability of Na^+^ and Ca^2+^ entry into the cells, finally leading to further depolarization [111]. Eventually, depolarization of the membrane is caused by these two phenomena, leading to the opening of voltage-dependent T-type calcium (Ca^2+^) and sodium (Na^+^) channels [112]. Entry of both ions facilitates enhanced membrane depolarization which, in turn, opens the voltage-dependent calcium channel, resulting in an increased intracellular concentration of Ca^2+^ ions. This increase in Ca^2+^ further leads to the fusion of secretory granules harboring insulin in the plasma membrane, and in this way, the first phase of insulin secretion is completed [113,114].

**Figure 6:**
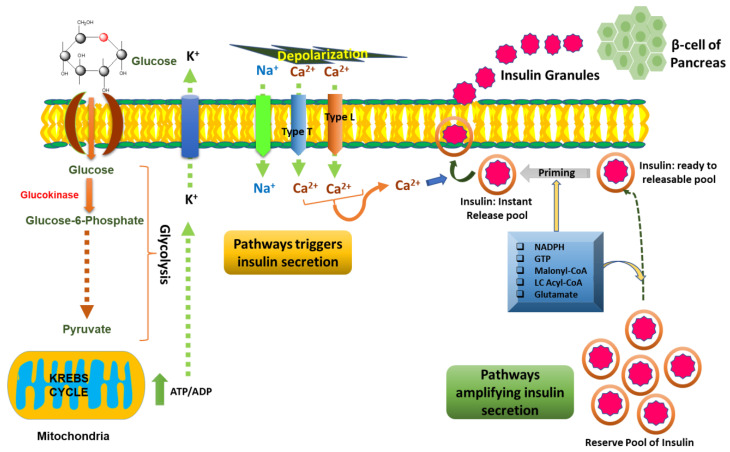
Pathways and mechanism of biphasic glucose-induced insulin secretion in the cell.

Apart from maintaining the ATP/ADP ratio in the cell, glucose metabolism is also involved in the regulation of many metabolic coupling signals which can initiate and eventually sustain a second phase of insulin secretion. NADPH, pyruvate, malate, citrate, isocitrate, acetyl-CoA and glutamate significantly play key roles in the regulation of insulin secretion [115]. Various other signaling pathways such as CaMKII, PKA, PKC and PKG also significantly facilitate glucose-mediated insulin secretion [116,117,118,119]. Figure 6 demonstrates the pathways involved in glucose-induced insulin secretion in the cell.

Unquestionably, insulin secretion is caused by pancreatic β-cells in response to elevated levels of nutrients (predominantly glucose) in the blood, and a number of signaling pathways are involved in it; however, these cells are very sensitive towards free radical species. The reason behind this sensitivity is the inefficient enzymatic antioxidant defensive system in pancreatic β-cells in comparison to other tissues such as liver tissue [120]. It is now established that hyperglycemia and oxidative stress are interlinked, and that ROS/RNS aid in diabetes progression. ROS aggravate the stress signaling pathways (NF-κB) in β-cells of the pancreas which results in β-cell apoptosis [121]. Oxidative stress also inhibits the respiratory chain in pancreatic β-cells, leading to suppression of glucose-mediated insulin secretion via a reduction in ATP generation [122,123]. Interestingly, insulin gene expression in pancreatic β-cells of db/db mice is modulated by oxidative stress by suppressing the level of the MafA and PDX-1 transcription factors [124]. Hyperglycemia also leads to excessive mitochondrial metabolism in β-cells, leading to alterations in the mitochondrial shape, volume and behavior as well as K-ATP channels, thus causing obstruction in glucose-mediated insulin secretion [125]. In spite of the numerous pieces of evidence of the close association of ROS and diabetes, the role of OS in the progression of diabetes is still enigmatic and skeptical, which needs to be further investigated.

## 6. Crucial Role of Nrf2 in OS-Induced Diabetes

The transcription factor nuclear factor erythroid-2-related factor 2 (also known as NRF2 or NFE2L2) is an important regulator of oxidative stress and has anti-inflammatory effects. This transcription factor drives over 50 redox homeostasis-related genes and nearly 200 genes influencing metabolism and repair [126]. Nrf2 is significantly involved in protection and helps the cells to combat environmental stress. Kelch-like erythroid-derived cap-n-collar homology factor associated protein I (Keap 1) is known as a negative regulator of Nrf2. Nrf2 and Keap1 play crucial roles in cellular redox signaling and have been studied extensively by various groups [127]. While Nrf2 is spontaneously expressed in the cytoplasm under normal physiological conditions, it is simultaneously repressed by Keap1. Translocation of Nrf2 into the nucleus is triggered by free radicals and other cellular stress conditions which lead to an increased expression of antioxidant proteins. The involvement of Nrf2 is also observed in many chronic ailments associated with OS and inflammation such as neurodegenerative diseases, vascular and metabolic diseases and diabetes. Recently, Nrf2 and Nrf2 activators have drawn the attention of scientists to exploit them for the treatment and prevention of diabetes and its complications.

Owing to its involvement in cytoprotection by inducing antioxidant and drug-metabolizing enzyme genes, the Nrf2-Keap1 system is extensively studied for its key role in diabetes. Yagishita et al. reported that Nrf2 plays a role in the protection of pancreatic β-cells from oxidative and nitrosative stress in a diabetic mouse model [128]. It was demonstrated that under the influence of ROS/RNS-induced stress conditions, transgenic mouse lines overexpressed inducible NO synthase (iNOS), particularly in pancreatic β-cells. Inducing Nrf2 in cell lines suppressed ROS/RNS levels significantly and hampered the β-cell damage. Nrf2 not only suppresses ROS/RNS-mediated damage but also plays a key role in the protection of pancreatic β-cells against arsenite-mediated damage [129]. Glucose homeostasis, as well as an enhancement in insulin sensitivity, is achieved by activation of Nrf2 to protect β-cells [130].

Experimental as well as computational models are extensively employed to investigate the role of Nrf2 in diabetes. Nrf2 activation by pterostilbene impaired oxidative stress, and pro-inflammatory cytokine toxicity via modulating the Nrf2 signaling network has been well studied [131,132,133,134]. A growing number of studies demonstrated that many Nrf2 activators such as resveratrol [135], sulforaphase [136], curcumin [137], quercerin [138] and CDDO (2-cyano-3,12-dioxooleana-1,9(11)-dien-28-oic acid) [139] have a positive impact on the protection of pancreatic β-cells. These compounds not only protect pancreatic β-cells but also assist in regaining their function against OS-mediated apoptosis and necrosis. The crucial involvement of Nrf2 is well documented in triggering series of genes such as heme oxygenase-1 (HO-1), superoxide dismutase (SOD), NAD(P)H quinone oxidoreductase (NQO1) and glutathione S-transferase (GST), which are significantly involved in antioxidant activity, detoxification, cellular redox homeostasis, glutathione homeostasis, etc. [131,132]. Nrf2 emerged as a promising therapeutic target to treat diabetes complications via its activation by the use of potential activators. A collection of findings indicates that the upregulation of Nrf2-dependent phase 2 genes protects cells, animals and humans against a wide variety of damaging molecules, including ROS and RNS, carcinogens, other electrophiles and radiation [140].

## 7. Summary

Undeniably, owing to its involvement in various health complications, OS has become a major theme of research worldwide. OS specifically deals with the redox imbalance of cells, which results in a malicious effect on membranes and biomolecules such as DNA, proteins and lipids. It is found to play a crucial role in two major mechanisms leading to diabetic complications, namely, insulin secretion and insulin action [2]. OS not only promotes the onset of diabetes but is also substantially involved in aggravating diabetes. It has been demonstrated that ROS are majorly involved in the impairment of β-cell function caused by the autoimmune response, cytokines and inflammatory proteins in type 1 DM [22]. OS has also been found to crop up by de novo generation of free radical species along with dysregulation of the antioxidant defense system in hyperglycemia [141]. It is well known that diabetes behaves differently towards the sensitivity to ROS, i.e., type 1 DM, which can be characterized by inflammatory damage, is significantly mediated by islet ROS, whereas a high nutrient flux and substantial ROS production mediate the loss of β-cell function in type 2 DM. ROS production is crucially implicated in the mitochondrial dysfunction and insulin resistance. ROS/RNS are extensively produced in the electron transport chain and mitochondria owing to nutrient overload, leading to O_2_^−^ overproduction [142]. ROS/RNS elevation is substantially involved in the formation and expression of AGE receptors, activation of the polyol pathway and PKC isoforms and upregulation of the hexosamine pathway which, in turn, lead to morbidity and worsening of type 2 DM. Concisely, it can be said that ROS/RNS produced by mitochondria aggravate the progression of type 2 DM [143,144,145].

## 8. Outlook and Future Perspective

The aim of this review was to highlight advances in understanding the role of metabolite-generated ROS in the development of diabetic complications and regulators of cellular defense mechanisms that manage chemical and oxidative stress. The rapid growth of the global population with diabetes, in recent years, has reached epidemic proportions. It is playing a significant role in morbidity, mortality and the economic cost to the society. Population growth, increases in obesity, aging, urbanization and lifestyle have added to diabetes complications. It is well documented that oxidative stress is substantially involved in the progression and development of diabetes and its complications. The increased oxidative stress in persons with type 2 DM is a consequence of several abnormalities, including hyperglycemia, insulin resistance, hyperinsulinemia and dyslipidemia.

A panoply of reports indicate that diabetes is centrally linked to metabolic disorders involving the overproduction of mitochondrial superoxides, consequently mediating diabetes-associated tissue damage [2]. The involvement of OS plays a pivotal role in insulin secretion dysfunction, as well as resistance, ultimately leading to diabetic complications.

Though an optimum level of ROS/RNS is required for the proper functioning of the cellular machinery and cell signaling in myriad ways, an elevation in ROS/RNS has a destructive effect. As free radical species are generated in oxygen-rich environments, cells usually have their own mechanism to combat against OS and defend themselves from ROS/RNS toxicity. Accumulating evidence has suggested that low concentrations of ROS/RNS are required for the signaling process triggering GSIS, whereas an elevated level has been suggested in the impairment of pancreatic β-cells [146]. ROS-mediated impairment of β-cell function is linked with a reduced level of the transcription factors Pdx-1 and MafA. These transcription factors are implicated in reduced insulin secretion as well as insulin levels by downregulating insulin gene expression. Oxidative stress has been shown to hamper/obstruct Pdx-1 and MafA expressions, ultimately leading to an improper functioning of these transcription factors [147,148,149].

Antioxidant enzymes, already present in the cellular environment, play crucial roles in ROS scavenging and maintenance of cellular redox homeostasis, resulting in a reduction in oxidative stress-mediated cell damage. Enzymatic antioxidants such as SOD, CAT, GR and GPX as well as non-enzymatic antioxidants such as vitamins E and C, GSH and GSSH were found to be key players in scavenging ROS/RNS. Although the role of antioxidant therapy in the pathophysiology of diabetes complications is still uncertain, it is understood that vitamin supplementation assists in the amelioration of oxidative stress. Since the Keap1-Nrf2-ARE pathway represents one of the most important cellular defense mechanisms against oxidative stress and xenobiotic damage, it has become an attractive target. Thus, targeting Nrf2/Keap1 pathways via small molecules would help in the prevention and treatment of oxidative stress-related diseases such as DM. More studies are required to substantiate the role of Nrf2-associated pathways in alleviating diabetic complications [150]. A natural alkaloid, berberine, has recently been reported to substantially reduce OS and inflammation by targeting several signaling pathways such as NF-κB, AMPK, Nrf2/HO and MAPKs in cells for the treatment of DM [151,152]. Thus, the activation of Nrf2 by natural compounds might prove a promising approach in the prevention of the hyperglycemia-induced oxidative stress [153]. It is also expected that Nrf2-interacting nutrients can re-balance insulin resistance. However, it is regrettable that despite the enormous advancement in science, some mysteries related to the role of ROS/RNS in type 2 diabetes are still unexplored. In future, therapies, vitamin supplementation, dietary habits and lifestyle changes in combination can be adopted for the treatment and prevention of diabetic complications.

## Figures and Tables

**Table 1:** Model subjects and experimental evidence to prove the role and effect of OS on organs.

Organism	Antioxidants and Biomolecules	Elevation/Reduction in Biomarkers for Oxidative Stress	Target Tissue/Organ Affected by Oxidative Stress	References
Animals	Enzymatic antioxidants	SOD ↓ CAT ↓ GR ↓ GPX ↓	Liver, Pancreas Liver Kidney	[53,54,55]
	Non-enzymatic antioxidants	Vit E ↓ Vit C ↓	Liver Kidney	[55]
		GSH ↓ GSSH ↓ GSH/GSSH ↓	Retina Heart Kidney, Hippocampus	[56] [57] [58,59]
	Lipids	TBARS ↑ Lipid peroxide ↑ MDA ↑	Kidney Kidney Kideny	[60,61]
	DNA	8-OHG ↑ 8-OHDG ↑	Plasma Liver, Kidney	[62,63]
	Proteins	Nitrotyrosine ↑	Retina Kidney	[56] [64]
	Reactive oxygen species/reactive nitrogen species	ROS/RNS ↑	Hippocampus	[59]
Humans	Enzymatic antioxidants	SOD ↑ CAT ↑ GPX ↑	Erythrocyte	[65]
	Non-enzymatic antioxidants	GSH ↓	Erythrocyte	[66]
	Lipid	MDA ↑ F2-isoprostanes ↑	Erythrocyte Urine	[67] [68]
	DNA	8-OHDG ↑	Urine	[69,70]
	Protein	Nitrotyrosine ↑ Protein carbonyl ↑	Plasma	[71] [72]

## Data Availability

Not applicable.
